# Clinical characteristics and outcomes of neonatal heart failure: a single-center retrospective analysis

**DOI:** 10.3389/fped.2026.1929001

**Published:** 2026-08-26

**Authors:** Sihan Wang, Lili Ma, Liangliang Li, Xiangyun Yin, Ping Yang, Xianghong Li, Hongmin Xi

**Affiliations:** Neonatology Department, The Affiliated Hospital of Qingdao University, Qingdao, China

**Keywords:** etiology, heart failure, mortality, neonate, risk factor analysis

## Abstract

**Background:**

Neonatal heart failure (HF) is a severe condition associated with high mortality because of immature organ function and limited cardiovascular reserve. However, studies focusing specifically on HF in neonates remain limited. This study aimed to characterize the clinical features of neonatal HF and identify factors associated with mortality, with additional analyses according to etiology and gestational age.

**Methods:**

This retrospective study included 89 neonates diagnosed with HF and admitted to our hospital between June 2016 and December 2025. Patients were classified into survival and death groups and were further stratified by etiology and gestational age (<34 weeks vs. ≥ 34 weeks). Logistic regression analyses were performed to identify factors associated with mortality, and model discrimination was evaluated using receiver operating characteristic (ROC) analysis.

**Results:**

The overall mortality was 31.5%. The primary causes of HF were critical congenital heart disease (28%), severe arrhythmia (13%), pulmonary hypertension (12%), extremely severe anemia (12%), and perinatal asphyxia (10%). Compared with the survival group, the death group had lower birth weight, lower Apgar scores, higher N-terminal pro-brain natriuretic peptide (NT-proBNP) levels, lower left ventricular ejection fraction (LVEF), and higher rate of fetal distress, respiratory failure, and shock. Multivariable analysis showed that fetal distress (OR, 7.70) and oliguria (OR, 7.32) were associated with higher mortality, whereas higher LVEF (OR per 1% increase, 0.94) was associated with lower mortality. The combined model had an AUC of 0.806 (95% CI, 0.702–0.909). In subgroup analysis, neonates born at <34 weeks had higher NT-proBNP levels, longer durations of invasive and non-invasive mechanical ventilation, longer hospital stays, higher rates of fetal distress, bronchopulmonary dysplasia and anemia, greater use of pulmonary surfactant, and higher mortality than those born at ≥34 weeks. No significant interactions by gestational age were observed for fetal distress, oliguria, or LVEF.

**Conclusions:**

Neonatal HF is associated with substantial mortality and is most commonly attributable to critical congenital heart disease, severe arrhythmia and other underlying conditions. Fetal distress and oliguria were independently associated with higher mortality, whereas higher LVEF was associated with lower mortality. Early recognition and targeted management are particularly important in preterm and other high-risk neonates.

## Introduction

Heart failure (HF) remains a major challenge in pediatric medicine, with representing a leading cause of early mortality in children, especially in neonate (1, 2). Epidemiological studies have reported an HF incidence of 1–9 per 1,000 person-years, whereas the global incidence of pediatric heart failure (PHF) is estimated at 0.87–7.4 per 100,000 children, with a mortality of approximately 6.3%–7% (3). Recent data indicate that the prevalence of PHF increased by 8.78% between 1990 and 2021 (4). Because pediatric HF is less common than adult HF and has heterogeneous pathophysiology, research on children remains limited, specifically in neonates. Emerging evidence also highlights substantial differences between pediatric and adult HF in underlying mechanisms, clinical presentation, and prognosis (5).

Advances in neonatal medicine, including surfactant replacement therapy and optimization of respiratory support, have substantially reduced mortality from neonatal respiratory failure (6–8). In contrast, neonatal circulatory management, particularly the recognition and treatment of HF, remains relatively underemphasized. The causes of neonatal HF are complex and heterogeneous, and structural congenital heart disease (CHD) is widely recognized as the most common cause (9). However, comprehensive analyses of factors associated with poor outcomes in neonatal HF remain scarce. Previous pediatric studies have identified age <30 days, CHD, ventricular tachycardia, vasoactive drug use, sepsis, mechanical ventilation, extracorporeal membrane oxygenation, pulmonary hypertension, and cardiac arrest as predictors of mortality (10). In neonates, myocardial immaturity and the transition from fetal to postnatal circulation may lead to delayed or maladaptive cardiovascular transition, persistent ductal patency, and consequent hemodynamic instability. Physiological differences across gestational ages may also influence the clinical course of HF. To date, few studies have specifically examined mortality-related factors in neonatal HF.

This retrospective study analyzed neonates with HF admitted to our center over the past decade to characterize the etiologic spectrum, compare clinical features across gestational-age strata, and identify factors associated with mortality. Given the limited data available regarding the causes and outcomes of neonatal HF, a better understanding of these factors is critical for optimizing management strategies and improving clinical outcomes. We hope that our findings will enhance awareness of neonatal HF and provide guidance for its early identification and clinical management.

## Method

### Study design and population

Neonates diagnosed with HF admitted to the Neonatal Intensive Care Unit (NICU) of the Affiliated Hospital of Qingdao University between June 2016 and December 2025 were included. The study was approved by the Institutional Review Board (Ethics Approval No. QYFYWZLL50261), and the requirement for informed consent was waived because of the retrospective design. Inclusion criteria were as follows: (1) term neonates within 28 days after birth or preterm neonates within 44 weeks of corrected gestational age; (2) fulfillment of the diagnostic criteria for neonatal HF. Patients with incomplete medical records or unclear treatment information were excluded. Patients were classified into survival and death groups according to in-hospital outcome, and the risk factors for mortality in children with heart failure were analyzed. To address etiological heterogeneity, patients were additionally classified into congenital-cause and non-congenital-cause groups according to their primary etiological. Clinical characteristics, echocardiographic findings, and in-hospital outcomes were compared between the two groups. In this cohort, congenital causes include congenital heart disease, congenital cardiomyopathy, and chromosomal or genetic abnormalities. Patients were also stratified by gestational age (<34 weeks vs. ≥ 34 weeks) to assess differences in clinical features and outcomes.

### Variables

Medical records were reviewed to collect demographic characteristics (sex, gestational age, birth weight, mode of delivery, and maternal comorbidities), clinical information (symptoms, laboratory tests, and imaging findings), treatment data, and outcomes. Routine blood and biochemical tests were performed on admission. Bedside echocardiography was performed using a Philips, EPIQ 7C ultrasound system and repeated as clinically indicated. Recorded echocardiographic parameters included cardiac chamber dimensions, LVIDd, IVSd, LVPWd, main pulmonary artery diameter, congenital cardiac abnormalities and shunt characteristics, left ventricular ejection fraction (LVEF), transmitral E/A ratio, tissue Doppler e′ velocity, TAPSE, valvular regurgitation, inferior vena cava diameter and respiratory variation, estimated right atrial pressure, and pulmonary artery systolic pressure. N-terminal pro-brain natriuretic peptide (NT-proBNP) measurement and echocardiography were performed within 24 h when HF was strongly suspected, with additional assessments performed as clinically indicated. For analysis, NT-proBNP and echocardiographic values corresponding to the most severe stage of HF during hospitalization were selected. All children received active etiological treatment, and vasoactive drugs were administered according to the underlying cause. Treatment response was assessed using clinical findings and echocardiography.

### Diagnostic criteria

Neonatal HF was diagnosed based on the overall clinical presentation and echocardiographic evidence, supplemented by the modified Ross score (11, 12). In neonates aged ≥4 postnatal days, HF was diagnosed using the modified Ross score. In neonates aged <4 postnatal days, an NT-proBNP level >5,309 pg/mL was considered supportive evidence of HF, and the initial diagnosis was based on the overall clinical presentation and echocardiographic evidence (13). NT-BNP levels are reassessed at 72 h post-onset to confirm the diagnosis and evaluate the condition according to the modified Ross score (Table 1).

**Table 1:** Modified ross score for infants aged 0∼3 months.

	score		
	0	1	2
Feeding (oz)	＞3.5	2.5–3.5	＜2.5
Timefor feeding (min)	＜20	20–40	＞40
Breathing	NL	Tachypnea	Retractions
RR/min	＜50	50–60	＞60
HR/min	＜160	160–170	＞170
Perfusion	NL	Reduced	Shocky
Hepatomegaly (cm)	＜2	2–3	＞3
NT-proBNP (pg/mL)	＜450	450–1,700	＞1,700
EF%	＞50	30–50	＜30
AV insufficiency	None	Mild	Moderate/severe

Total score: 0–5 (no HF), 6–10 (mild HF), 11–15 (moderate HF), 16–20 (severe HF). A score of ≥6 points is diagnosed as heart failure. NT-proBNP measurement is required 4 days after birth. Oz: ounce; Nl: normal; RR: respiratory rate; HR: heart rate; NT-proBNP: N-terminal pro-brain natriuretic peptide; EF: ejection fraction; AV: systemic atrio ventricular valve.

Oliguria was defined as urine output <1.0 mL/kg/h (14). Fetal distress was diagnosed using predefined clinical and ultrasonographic criteria. Clinical criteria included an abnormal fetal heart rate (>160 or <110 beats/min in the absence of uterine contractions or fetal movement), decreased fetal movement (<10 movements/12 h), excessive fetal movement (>10 movements/h), meconium-stained amniotic fluid, or abnormal fetal blood gas values (pH <7.20, PO₂ < 10 mmHg, or PCO₂ > 60 mmHg). Ultrasonographic criteria included an abnormal fetal heart rate or movement, sonographic findings suggestive of meconium-stained amniotic fluid, a nuchal cord accompanied by abnormal umbilical artery Doppler findings (S/D ratio >3 or resistance index >0.6), or oligohydramnios (maximum vertical pocket ≤20 mm) (12, 15).

Intrauterine infection means chorioamnionitis, including clinical chorioamnionitis and histological chorioamnionitis. Clinical chorioamnionitis was diagnosed when maternal fever during labor was ≥39.0 °C, or 38.0–38.9 °C persisting for >30 min, together with at least one of the following: (1) maternal white blood cell count >15   ×   10⁹/L in the absence of corticosteroid treatment; (2) Definite purulent fluid from the cervical os; (3) Baseline fetal tachycardia >160 beats/min for ≥10 min, excluding accelerations, decelerations, and periods of marked variability; (4) Turbid or foul-smelling amniotic fluid. Histological chorioamnionitis was diagnosed by pathological examination of the umbilical cord, placenta, or fetal membranes showed evidence of inflammation or infection (16).

Early-onset sepsis (EOS) was defined as blood culture-confirmed sepsis occurring within the first 3 days after birth. Late-onset sepsis (LOS) was defined as blood culture-confirmed sepsis occurring >3 days after birth (17).

Bronchopulmonary dysplasia (BPD) was defined as a requirement for supplemental oxygen or respiratory support for at least 3 consecutive days at 36 weeks' postmenstrual age in preterm infants born at <32 weeks of gestation, together with persistent radiographic evidence of parenchymal lung abnormalities (18).

### Statistical analysis

Statistical analyses were performed using SPSS version 26.0. Normally distributed continuous variables are presented as mean ± standard deviation and were compared using the independent-samples t test. Skewed continuous variables are presented as median (interquartile range) and were compared using a rank-sum test. Categorical variables are presented as n (%) and were compared using the chi-square test, continuity-corrected chi-square test, or Fisher's exact test, as appropriate. Univariable and multivariable logistic regression analyses were used to evaluate factors associated with in-hospital mortality. A parsimonious multivariable model was constructed, and model discrimination was assessed using ROC curves and AUCs with 95% CIs. Subgroup analyses by etiology and gestational age used Firth logistic regression, with interaction terms used to assess effect modification. LVEF was analyzed per 5% increase in subgroup analyses. Missingness was <5% for all variables included in the analyses, and missing values were not imputed. A two-sided *P* < 0.05 was considered statistically significant.

## Results

### Baseline characteristics of the study population

#### General information

A total of 89 neonates with HF were included, comprising 53 males (59.6%) and 36 females (40.4%). The proportion of females was higher in the death group (*P* = 0.030). The median gestational age was 37.0 weeks (IQR, 34.4–38.9), and the median birth weight was 2,800 g (IQR, 2,100–3,250). Compared with survivors, non-survivors had a lower birth weight (*P* = 0.004), a trend toward lower gestational age (*P* = 0.085), and lower 1- and 5-minute Apgar scores (both *P* < 0.05). The median age at HF onset was 1 day in the death group and 2 days in the survival group, with no significant between-group difference (*P* = 0.446). Fetal distress was more common among non-survivors (*P* < 0.001). No maternal comorbidity differed significantly between the two groups (Table 2).

**Table 2:** General information in the two groups.

Variables	Survival group (*n* = 61)	Death group (*n* = 28)	Statistic	*P*
gestational age (weeks), M (Q₁, Q₃)	37.60 (35.40, 38.60)	34.80 (28.55, 39.32)	Z = −1.72	0.085
birth weight (grams), M (Q₁, Q₃)	2,920.00 (2,340.00, 3,330.00)	2,295.00 (965.00, 2,925.00)	Z = −2.85	0.004*
1 min Apgar scores, M (Q₁, Q₃)	10.00 (8.00, 10.00)	6.50 (3.00, 10.00)	Z = −3.38	<.001*
5 min Apgar scores, M (Q₁, Q₃)	10.00 (9.00, 10.00)	8.00 (5.00, 10.00)	Z = −2.67	0.008*
HF onset age (days), M (Q₁, Q₃)	1.00 (1.00, 6.00)	2.00 (1.00, 7.75)	Z = −0.76	0.446
boy, n(%)	41 (67.21)	12 (42.86)	*χ*^2^ = 4.73	0.030*
gestational diabetes, n(%)	15 (24.59)	5 (17.86)	χ^2^ = 0.50	0.480
gestational hypertension, n(%)	8 (13.11)	4 (14.29)	χ^2^ = 0.00	1.000
placental abnormalities, n(%)	4 (6.56)	3 (10.71)	χ^2^ = 0.06	0.801
maternal immune-related diseases, n(%)	3 (4.92)	3 (10.71)	χ^2^ = 0.31	0.577
fetal distress, n(%)	10 (16.39)	18 (64.29)	χ^2^ = 20.41	<.001*
intrauterine infection, n(%)	17 (26.23)	5 (17.86)	-	0.610
EOS, n(%)	16 (26.23)	10 (35.71)	χ^2^ = 0.83	0.361
LOS, n(%)	3 (4.92)	4 (14.29)	χ^2^ = 1.21	0.271
anemia, n(%)	21 (34.43)	13 (46.43)	χ^2^ = 1.17	0.279

*Statistical significance (*P* < 0.05) in test.

HF, heart failure; EOS, early-onset sepsis; LOS, late-onset sepsis.

#### Clinical manifestations and auxiliary examinations of heart failure

Respiratory failure and shock occurred in 92.86% and 64.29% of non-survivors, respectively, compared with 70.49% and 32.79% of survivors (*P* = 0.019 and *P* = 0.005, respectively). Oliguria was observed in 32.1% of the death group compared with 6.6% in survivors (*P* = 0.004). In the death group, the median NT-proBNP level was 32,137.00 pg/mL and the median LVEF was 55%, which had lower LVEF (*P* = 0.040), and higher NT-proBNP levels (*P* = 0.017). Cardiomegaly was observed in 70.8% of neonates, 38.2% had atrial septal defects, 21.4% had ventricular septal defect, 4.5% experienced ventricular tachycardia, and 11.2% experienced supraventricular tachycardia. None of these findings differed significantly between the survival and death groups (Table 3).

**Table 3:** Clinical manifestations and auxiliary examinations of HF in the two groups.

Variables	Survival group (*n* = 61)	Death group (*n* = 28)	Statistic	*P*
size of ASD (cm), M (Q₁, Q₃)	0.00 (0.00, 0.30)	0.00 (0.00, 0.39)	Z = −0.60	0.551
size of VSD (cm), M (Q₁, Q₃)	0.00 (0.00, 0.00)	0.00 (0.00, 0.00)	Z = −0.35	0.725
size of PDA (cm), M (Q₁, Q₃)	0.19 (0.00, 0.32)	0.20 (0.00, 0.31)	Z = −0.67	0.501
LVEF (%), M (Q₁, Q₃)	60.00 (52.00, 66.00)	55.00 (43.75, 61.75)	Z = −2.05	0.040*
NT-proBNP, M (Q₁, Q₃)	11,860.50 (3,694.00, 22,868.00)	32,137.00 (5,000.00, 35,000.00)	Z = −2.40	0.017*
pulmonary arterial pressure (mmHg), M (Q₁, Q₃)	50.00 (39.00, 55.00)	46.00 (38.75, 55.25)	Z = −0.70	0.484
shock, n(%)	20 (32.79)	18 (64.29)	χ^2^ = 7.78	0.005*
Cardiomegaly, n(%)	43 (70.49)	20 (71.43)	χ^2^ = 0.01	0.928
left ventricle enlargement, n(%)	5 (8.20)	7 (25.00)	χ^2^ = 3.32	0.069
right ventricle enlargement, n(%)	26 (42.62)	6 (21.43)	χ^2^ = 3.74	0.053
fully ventricle enlargement, n(%)	13 (21.31)	8 (28.57)	χ^2^ = 0.56	0.454
ASD, n(%)	22 (36.07)	12 (42.86)	χ^2^ = 0.37	0.540
VSD, n(%)	14 (22.95)	5 (17.86)	χ^2^ = 0.30	0.586
ventricular tachycardia, n(%)	3 (4.92)	1 (3.57)	χ^2^ = 0.00	1.000
supraventricular tachycardia, n(%)	9 (14.75)	1 (3.57)	χ^2^ = 1.42	0.234
PPHN, n(%)	52 (85.25)	24 (85.71)	χ^2^ = 0.00	1.000
respiratory failure, n(%)	43 (70.49)	26 (92.86)	χ^2^ = 5.51	0.019*
oliguria, n(%)	4 (6.56)	9 (32.14)	χ^2^ = 8.13	0.004*

*Statistical significance (*P* < 0.05) in test.

ASD, atrial septal defect; VSD, ventricular septal defect; PDA, patent ductus arteriosus; LEVF, left ventricular ejection fraction; NT-proBNP, N-terminal pro-brain natriuretic peptide; PPHN, persistent pulmonary hypertension of the newborn.

#### Therapy

All neonates received standard supportive care. Dopamine was administered to 61.8% of patients, epinephrine to 28.1%, and digoxin to 20.2%. Dopamine (*P* = 0.007) and epinephrine (*P* = 0.009) were used more frequently among the death group, whereas digoxin was used more frequently among survivors (*P* = 0.008). The duration of invasive mechanical ventilation was longer in the death group (*P* = 0.003) (Table 4).

**Table 4:** Therapy in the two groups.

Variables	Survival group (*n* = 61)	Death group (*n* = 28)	Statistic	*P*
invasive ventilator ventilation time (days), M (Q₁, Q₃)	0.00 (0.00, 4.00)	2.50 (1.00, 9.25)	Z = −2.98	0.003*
noninvasive ventilation time (days), M (Q₁, Q₃)	3.00 (0.00, 7.00)	0.00 (0.00, 3.50)	Z = −1.58	0.113
oxygen therapy time (days), M (Q₁, Q₃)	6.00 (2.00, 21.00)	6.00 (2.00, 10)	Z = 0.74	0.462
dopamine, n(%)	32 (52.46)	23 (82.14)	χ^2^ = 7.16	0.007*
epinephrine, n(%)	12 (19.67)	13 (46.43)	χ^2^ = 6.80	0.009*
digoxin, n(%)	17 (27.87)	1 (3.57)	χ^2^ = 7.02	0.008*
antiarrhythmic drugs, n(%)	10 (16.39)	3 (10.71)	χ^2^ = 0.15	0.703
cardiac surgery, n(%)	6 (9.84)	3 (10.71)	χ^2^ = 0.00	1.000
PS, n(%)	21 (34.43)	14 (50.00)	χ^2^ = 1.95	0.163
drugs for PPHN, n(%)	6 (9.84)	3 (10.71)	χ^2^ = 0.00	1.000
iNO, n(%)	5 (8.20)	5 (17.86)	χ^2^ = 0.96	0.328

*Statistical significance (*P* < 0.05) in test.

PS, pulmonary surfactant; PPHN, persistent pulmonary hypertension of the newborn; iNO, inhalable Nitric Oxide.

#### Outcome and prognosis

In our cohort, the mortality rate among the 89 neonates with HF was 31.5%, reflecting the severity of neonatal HF. The average survival duration was 7 days for neonates who died. The median time from HF diagnosis to death was 3.50 days (IQR, 0.00–14.25 days). Of the 28 deaths, 15 (53.6%) were associated with cardiac causes, including congenital heart disease, arrhythmias, cardiomyopathy, and cardiac tamponade, whereas 13 (46.4%) were associated with non-cardiac causes, including pulmonary hypertension, infection, severe anemia, perinatal asphyxia, and genetic abnormalities. Major complications included BPD (7.9%) and pulmonary hemorrhage; pulmonary hemorrhage occurred more frequently in the death group (*P* = 0.001). The incidence of intracranial hemorrhage did not differ significantly between groups (Table 5).

**Table 5:** Outcome and prognosis in the two groups.

Variables	Survival group (*n* = 61)	Death group (*n* = 28)	Statistic	*P*
Length of hospital stay (days), M (Q₁, Q₃)	17.00 (11.00, 28.00)	7.00 (2.00, 28.75)	Z = −2.57	0.010*
BPD, n(%)	3 (4.92)	5 (17.86)	χ^2^ = 2.51	0.113
pulmonary hemorrhage, n(%)	6 (9.84)	11 (39.29)	χ^2^ = 10.77	0.001*
Intracranial hemorrhage, n(%)	6 (9.84)	5 (17.86)	χ^2^ = 0.52	0.471

*Statistical significance (*P* < 0.05) in test.

BPD: bronchopulmonary dysplasia.

### Etiology of heart failure

In this cohort, the primary etiologies of neonatal heart failure were critical congenital heart disease (CCHD) (28%), severe arrhythmia (13%), pulmonary hypertension (12%), extremely severe anemia (12%), and perinatal asphyxia (10%). CCHD in the cohort included anomalous pulmonary venous drainage, truncus arteriosus, coarctation of the aorta, congenital endocardial cushion defect, large ventricular septal defect. Arrhythmias included supraventricular tachycardia, atrial flutter, atrial tachycardia, ventricular tachycardia, and conduction block. Other etiologies included sepsis (7%), chromosomal or genetic abnormalities (6%), cardiac tamponade (1%), hyperthyroidism (1%), myocarditis (1%), pneumothorax (1%), and others (8%). Among the five most common etiologies, CCHD accounted for the largest number of deaths (*n* = 9).

### Factors associated with death

**Figure 1:**
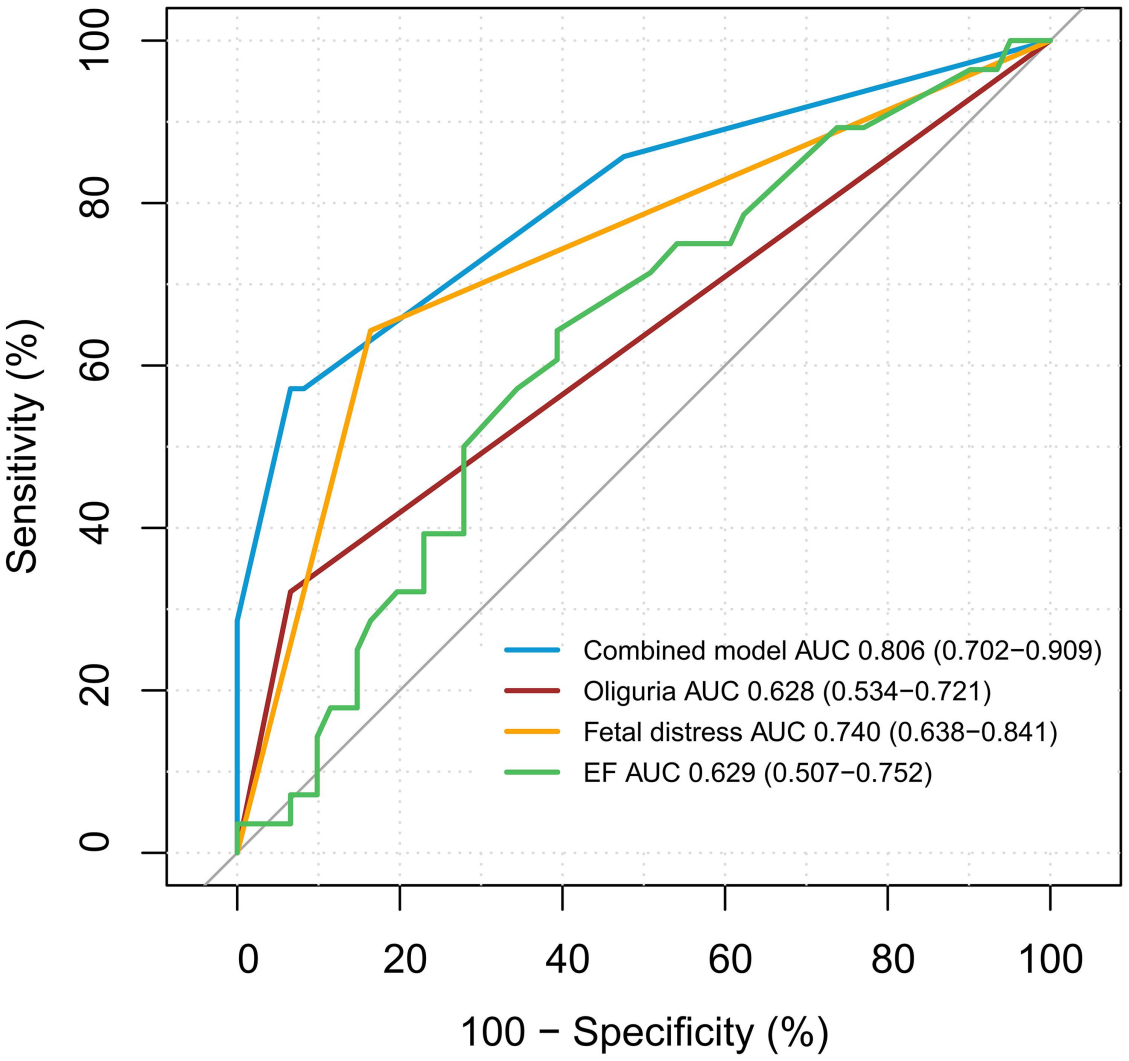
Receiver operating characteristic curves for the combined model and individual predictors of mortality in neonates with heart failure. The ROC curves show the discriminatory performance of the combined model and individual predictors for mortality. The combined model (fetal distress + oliguria + LVEF) achieved an AUC of 0.806 (95% CI, 0.702–0.909), compared with 0.740 (95% CI, 0.638–0.841) for fetal distress, 0.629 (95% CI, 0.507–0.752) for LVEF, and 0.628 (95% CI, 0.534–0.721) for oliguria. AUC, area under the curve; CI, confidence interval; LVEF, left ventricular ejection fraction; ROC, receiver operating characteristic.

Univariate analysis identified several variables associated with mortality. In multivariable logistic regression, male sex was inversely associated with mortality (OR, 0.21; 95% CI, 0.06–0.73; *P* = 0.014), and higher LVEF was associated with lower odds of mortality (OR, 0.94; 95% CI, 0.90–0.99; *P* = 0.028). Fetal distress (OR, 7.70; 95% CI, 2.17–27.24; *P* = 0.002), and oliguria (OR, 7.32; 95% CI, 1.40–38.17; *P* = 0.018) were associated with higher odds of death (Table 6). VIF values ranged from 1.121 to 3.738, indicating no substantial multicollinearity among the candidate variables. The combined model (fetal distress + oliguria + LVEF) had an AUC of 0.806 (95% CI, 0.702–0.909), indicating good apparent discrimination for mortality. Its AUC was numerically higher than that of fetal distress, oliguria, or LVEF alone (Figure 1).

**Table 6:** Univariate and multivariate logistic regression analysis of potential effect factors and death of neonate HF in the two groups.

Variables	Univariate analysis					VIF		Multivariate analysis				
	*β*	S.E	Z	*P*	OR (95%CI)			β	S.E	Z	*P*	OR (95%CI)
boys	−1.01	0.47	−2.14	0.032*	0.37 (0.15∼0.92)	1.162		−1.58	0.64	−2.45	0.014*	0.21 (0.06∼0.73)
fetal distress	2.22	0.52	4.23	<.001*	9.18 (3.28∼25.67)	2.403		2.04	0.64	3.16	0.002*	7.70 (2.17∼27.24)
shock	1.31	0.48	2.72	0.006*	3.69 (1.44∼9.44)	1.391						
respiratory Failure	1.69	0.79	2.16	0.031*	5.44 (1.17∼25.38)	1.146						
oliguria	1.91	0.66	2.91	0.004*	6.75 (1.86∼24.45)	1.220		1.99	0.84	2.36	0.018*	7.32 (1.40∼38.17)
gestational age	−0.17	0.06	−2.80	0.005*	0.84 (0.75∼0.95)	1.396						
1 min Apgar scores	−0.32	0.09	−3.57	<.001*	0.73 (0.61∼0.87)	3.738						
5 min Apgar scores	−0.23	0.09	−2.47	0.014*	0.79 (0.66∼0.95)	2.162						
LVEF	−0.03	0.02	−1.96	0.050	0.97 (0.93∼0.99)	1.205		−0.06	0.03	−2.20	0.028*	0.94 (0.90∼0.99)
BNP	0.00	0.00	1.68	0.092	1.00 (1.00∼1.00)	1.121						

*Statistical significance (*P* < 0.05) in Logistic regression analysis.

SE, standard error; OR, Odds Ratio; CI, Confidence Interval; LEVF, left ventricular ejection fraction; BNP: brain natriuretic peptide.

### Etiology-based subgroup analysis

The cohort comprised 60 neonates in the non-congenital-cause group and 29 in the congenital-cause group. Compared with the congenital-cause group, the non-congenital-cause group had higher rates of EOS (*P* = 0.026), shock (P＝0.014), respiratory failure (*P* = 0.015), and anemia (*P* < 0.001). In contrast, the congenital-cause group had a higher median pulmonary arterial pressure (*P* = 0.046) and a higher prevalence of ventricular septal defect (*P* < 0.001). No significant differences were observed in LVEF, NT-proBNP level, fetal distress, oliguria, or mortality between two groups. Mortality was 27.6% in the congenital-cause group and 33.3% in the non-congenital-cause group (*P* = 0.584) (Supplementary file 1). In the Firth regression analysis, fetal distress (*P* < 0.001) and oliguria (*P* = 0.010) were associated with mortality in the non-congenital-cause group, whereas neither showed a significant interaction with etiology (P for interaction=0.297 and 0.926, respectively). In contrast, LVEF showed a significant interaction (P for interaction = 0.014), with higher LVEF associated with lower mortality only in the congenital-cause group (Supplementary file 2).

### Gestational age subgroup analysis

Neonates were stratified into two gestational-age groups: <34 weeks (*n* = 20) and ≥34 weeks (*n* = 69). Infants <34 weeks had lower birth weights and Apgar scores, higher NT-proBNP levels, longer durations of invasive and noninvasive mechanical ventilation, and longer hospital stays. They also had higher rates of fetal distress, BPD, and anemia, a lower incidence of right heart enlargement, increased use of pulmonary surfactant, and higher mortality (all *P* < 0.05 for the reported differences) (Table 7). In Firth regression analyses, fetal distress was associated with mortality in both gestational-age groups (*P* = 0.006 and *P* = 0.011), whereas oliguria was associated with mortality only in the ≥34-week group (*P* = 0.035). LVEF was not significantly associated with mortality in either subgroup (*P* = 0.077 and *P* = 0.540). No significant interactions with gestational age were observed for fetal distress, oliguria, or LVEF (all P for interaction >0.05). (Supplementary file 3)

**Table 7:** Comparison of clinical information in the <34 weeks gestational age group and ≥34 weeks gestational age group.

Variables	≥34 weeks (*n* = 69)	<34 weeks (*n* = 20)	Statistic	*P*
birth weight, M (Q₁, Q₃)	3,000.00 (2,560.00, 3,350.00)	1,205.00 (915.00, 1,565.00)	Z = −6.49	<.001*
1 min Apgar scores, M (Q₁, Q₃)	10.00 (8.00, 10.00)	5.00 (3.00, 8.00)	Z = −4.39	<.001*
5 min Apgar scores, M (Q₁, Q₃)	10.00 (9.00, 10.00)	7.50 (5.00, 9.00)	Z = −3.53	<.001*
size of ASD, M (Q₁, Q₃)	0.00 (0.00, 0.35)	0.00 (0.00, 0.28)	Z = −0.39	0.694
size of VSD, M (Q₁, Q₃)	0.00 (0.00, 0.00)	0.00 (0.00, 0.00)	Z = −0.78	0.438
size of PDA, M (Q₁, Q₃)	0.20 (0.00, 0.32)	0.23 (0.00, 0.33)	Z = −0.83	0.407
LEVF, M (Q₁, Q₃)	60.00 (46.00, 65.00)	60.00 (55.00, 65.25)	Z = −0.67	0.503
BNP, M (Q₁, Q₃)	9,952.00 (3,890.67, 29,489.00)	32,137.00 (12,544.00, 32,852.75)	Z = −2.37	0.018*
pulmonary arterial pressure, M (Q₁, Q₃)	52.00 (45.00, 60.00)	36.50 (0.00, 44.50)	Z = −3.78	<.001*
invasive ventilator ventilation time, M (Q₁, Q₃)	1.00 (0.00, 5.00)	4.50 (1.75, 6.25)	Z = −2.04	0.041*
noninvasive ventilation time, M (Q₁, Q₃)	1.00 (0.00, 5.00)	7.00 (0.00, 22.25)	Z = −2.66	0.008*
HF onset age (day), M (Q₁, Q₃)	1.00 (1.00, 6.00)	1.50 (1.00, 7.50)	Z = −0.51	0.607
oxygen therapy time, M (Q₁, Q₃)	1.00 (1.00, 6.00)	1.00 (1.00, 4.00)	Z = −0.68	0.495
length of hospital stay, M (Q₁, Q₃)	13.00 (8.00, 24.00)	47.50 (9.25, 58.25)	Z = −3.03	0.002*
boys, n(%)	43 (62.32)	10 (50.00)	χ^2^ = 0.98	0.323
gestational diabetes, n(%)	15 (21.74)	5 (25.00)	χ^2^ = 0.00	0.997
gestational hypertension, n(%)	8 (11.59)	4 (20.00)	χ^2^ = 0.36	0.550
placental abnormalities, n(%)	5 (7.25)	2 (10.00)	χ^2^ = 0.00	1.000
maternal immune-related diseases, n(%)	3 (4.35)	3 (15.00)	χ^2^ = 1.36	0.243
fetal distress, n(%)	16 (23.19)	12 (60.00)	χ^2^ = 9.74	0.002*
intrauterine infection, n(%)	17 (24.64)	4 (20.00)	-	0.823
EOS, n(%)	19 (27.54)	7 (35.00)	χ^2^ = 0.42	0.518
LOS, n(%)	4 (5.80)	3 (15.00)	χ^2^ = 0.76	0.382
shock, n(%)	26 (37.68)	12 (60.00)	χ^2^ = 3.16	0.076
cardiomegaly, n(%)	52 (75.36)	11 (55.00)	χ^2^ = 3.11	0.078
left ventricle enlargement, n(%)	8 (11.59)	4 (20.00)	χ^2^ = 0.36	0.550
right ventricle enlargement, n(%)	31 (44.93)	1 (5.00)	χ^2^ = 10.73	0.001*
fully ventricle enlargement, n(%)	16 (23.19)	5 (25.00)	χ^2^ = 0.00	1.000
ASD, n(%)	26 (37.68)	8 (40.00)	χ^2^ = 0.04	0.851
VSD, n(%)	16 (23.19)	3 (15.00)	χ^2^ = 0.23	0.633
ventricular tachycardia, n(%)	3 (4.35)	1 (5.00)	-	1.000
supraventricular tachycardia, n(%)	8 (11.59)	2 (10.00)	χ^2^ = 0.00	1.000
PPHN, n(%)	63 (91.30)	13 (65.00)	χ^2^ = 6.62	0.010*
dopamine, n(%)	42 (60.87)	13 (65.00)	χ^2^ = 0.11	0.738
epinephrine, n(%)	18 (26.09)	7 (35.00)	χ^2^ = 0.61	0.435
digoxin, n(%)	15 (21.74)	3 (15.00)	χ^2^ = 0.12	0.730
antiarrhythmic drugs,n(%)	12 (17.39)	1 (5.00)	χ^2^ = 1.04	0.307
cardiac surgery, n(%)	6 (8.70)	3 (15.00)	χ^2^ = 0.16	0.688
respiratory failure, n(%)	50 (72.46)	19 (95.00)	χ^2^ = 3.32	0.068
BPD, n(%)	1 (1.45)	7 (35.00)	χ^2^ = 17.43	<.001*
anemia, n(%)	22 (31.88)	12 (60.00)	χ^2^ = 5.19	0.023*
oliguria, n(%)	7 (10.14)	6 (30.00)	χ^2^ = 3.44	0.064
pulmonary hemorrhage, n(%)	14 (20.29)	3 (15.00)	χ^2^ = 0.04	0.836
Intracranial hemorrhage, n(%)	6 (8.70)	5 (25.00)	χ^2^ = 2.45	0.118
PS, n(%)	19 (27.54)	16 (80.00)	χ^2^ = 17.89	<.001*
drugs for PPHN, n(%)	9 (13.04)	0 (0.00)	χ^2^ = 1.64	0.200
iNO, n(%)	8 (11.59)	2 (10.00)	χ^2^ = 0.00	1.000
death, n(%)	16 (23.19)	12 (60.00)	χ^2^ = 9.74	0.002*

*Statistical significance (*P* < 0.05) in test.

ASD, atrial septal defect; VSD, ventricular septal defect; PDA, patent ductus arteriosus; LEVF, left ventricular ejection fraction; BNP, brain natriuretic peptide; HF, heart failure; EOS, Early-onset sepsis; LOS, Late-onset sepsis; PPHN, persistent pulmonary hypertension of the newborn; BPD, bronchopulmonary dysplasia; PS, pulmonary surfactant; PPHN, persistent pulmonary hypertension of the newborn; iNO, inhalable Nitric Oxide.

## Discussion

Neonatal HF is a complex clinical syndrome in which a failure of the heart cannot provide sufficient cardiac output to meet systemic metabolic demands (19). Its pathophysiology differs from that of HF in older children and adults because neonates undergo rapid transition from fetal to postnatal circulation and have limited myocardial reserve. These developmental characteristics increase susceptibility to hypoxia, infection, pulmonary hypertension, and abrupt changes in volume or pressure load (20–24). Consequently, neonatal HF often reflects the interaction of multiple pathological processes rather than a single mechanism. Previous studies have reported the mortality of approximately 6.3%–7% in pediatric HF (3), whereas mortality reached 31.5% in our neonatal HF cohort. Although these estimates are not directly comparable because of differences in study populations and settings, the high mortality observed in our cohort underscores the severity of HF among hospitalized neonates.

The etiologies of neonatal HF are diverse and include CCHD as well as arrhythmias, sepsis, myocarditis, hypoxia, metabolic disturbances, and anemia (9, 25). In our cohort, the most common etiologies were CCHD (28%), arrhythmia (13%), pulmonary hypertension (12%), anemia (12%), and asphyxia (10%). CCHD is defined by systemic low cardiac output requiring surgery or catheter-based intervention during the first year of life. Rapid deterioration may occur after disease onset, and mortality during the first year of life has been reported to reach 25% (26). CCHD is also recognized as a leading cause of neonatal HF (20). Not all children with CCHD will develop heart failure; they must meet both the diagnostic criteria for CCHD and heart failure outlined above. Furthermore, children presenting solely with severe cyanosis, duct-dependent circulation, profound hypoxemia, metabolic acidosis, or shock, without accompanying clinical and echocardiographic evidence of heart failure, are insufficient to be classified as heart failure associated with congenital heart disease. CCHD accounted for the largest number of deaths in our cohort, highlighting the importance of structural hemodynamic abnormalities. Severe arrhythmias may precipitate HF by reducing effective cardiac output and should therefore be identified and treated promptly. These measures are particularly important in preterm or low-birth-weight infants, who have heightened vulnerability due to organ immaturity. Accordingly, the initial evaluation of a neonate with suspected HF should rapidly clarify the underlying mechanism, including structural heart disease, arrhythmia, pulmonary hypertension, infection, and anemia, because these conditions require substantially different management strategies.

LVEF is an important echocardiographic measure of left ventricular systolic function in neonates with HF (27). In our study, higher LVEF was associated with lower mortality, consisting with previous neonatal studies showing that lower LVEF is more common among non-survivors with severe birth asphyxia or hypoxic-ischemic encephalopathy (28). However, this finding should be interpreted cautiously because LVEF primarily reflects left ventricular systolic function and is influenced by loading conditions. Neonates with duct-dependent lesions, cyanotic CHD, abnormal shunting, or right ventricular dysfunction may retain preserved LVEF despite severe hemodynamic compromise. Therefore, preserved LVEF does not exclude clinically significant HF, and LVEF should be interpreted as part of a comprehensive hemodynamic assessment rather than as a universal prognostic marker.

Fetal distress was associated with higher mortality for neonatal HF in our study. Hypoxia may alter neonatal cardiovascular function, reduce myocardial oxygen delivery, and impair contractility, making the heart more prone to decompensation during circulatory transition (21, 22). Fetal distress is not only a cause of heart failure, but may also be an early clinical clue that fetal or neonatal cardiovascular compromise is already present. Previous studies have identified fetal compromise, progressive deterioration in cardiac performance, hydrops, and reduced cardiac output are important indicators of significant fetal circulatory dysfunction (29–31). Oliguria was also associated with mortality and may reflect impaired systemic and renal perfusion during circulatory compromise (32–34). A recent study of neonates with HF within the first 28 days of life found that adverse postnatal responses associated with asphyxia were independent predictors of mortality, indirectly indicating that fetal distress increases the risk of neonatal HF death. The study also identified oliguria as the strongest predictor of mortality, findings that are consistent with our results (35). From a practical perspective, a history of fetal distress should lower the threshold for cardiovascular assessment when a neonate develops respiratory instability, poor perfusion, arrhythmia, or other signs of circulatory dysfunction. Similarly, persistent oliguria should prompt reassessment of systemic perfusion, blood pressure, renal function, fluid balance, and cardiovascular status. Neither finding is specific to HF, but both may help identify neonates who require closer observation and earlier hemodynamic evaluation.

The etiology-stratified analysis further demonstrated the clinical heterogeneity of neonatal HF. Despite these in clinical profiles, mortality was similar between the congenital-cause and non-congenital-cause groups. The associations of fetal distress and oliguria with mortality were directionally consistent across etiological categories, and no significant interactions were detected. These findings suggest that perinatal compromise and systemic hypoperfusion may represent clinically relevant warning signs across different etiologies of neonatal HF. In contrast, the association between LVEF and mortality differed by etiological category. Because the sample size was reduced after stratification, this finding should be considered exploratory. Risk assessment should not rely solely on whether HF is congenital or non-congenital. Instead, clinical severity, organ perfusion, and the underlying hemodynamic abnormality should be considered together.

In our study cohort, neonates born at less than 34 weeks had less favorable clinical characteristics and outcomes than those born at ≥34 weeks. They had lower birth weight and Apgar scores, higher NT-proBNP levels, longer durations of mechanical ventilation and hospitalization, and higher mortality. These findings underscore the greater severity of heart failure in this population. The limited cardiovascular reserve associated with prematurity may reduce tolerance to hemodynamic stress and contribute to more complicated clinical courses (1, 36). In addition, preterm infants inherently have reduced respiratory reserve, so heart failure may increase the work of breathing, thereby prolonging the need for ventilatory support (37–39). Previous studies have shown that prematurity increases the risk of mortality in neonates with CHD (40), but direct comparisons of HF outcomes across gestational-age strata remain limited. By focusing specifically on neonates with HF and integrating echocardiographic and laboratory data with clinical outcomes, our study confirms that low gestational age not only increases mortality but is also associated with more severe acute decompensation. These results emphasize the need for gestational age–based risk stratification. Preterm neonates with HF may require closer monitoring, early supportive care, and careful adjustment of inotropic therapy and fluid management. Although mortality was substantially higher among neonates born at <34 weeks, interaction analyses did not show that the associations of fetal distress, oliguria, or LVEF with mortality differed significantly between gestational-age strata. Thus, the present data do not support gestational-age-specific effects of these mortality-associated factors. Because only 20 neonates were included in the <34-week subgroup, these findings should be considered exploratory.

Dopamine, epinephrine, and invasive mechanical ventilation were used more frequently in the death group. These differences likely reflect greater illness severity and the need for more intensive support rather than harmful effects of these therapies. Similarly, the more frequent use of digoxin among survivors should not be interpreted as evidence of a survival benefit because treatment selection was influenced by the underlying etiology and clinical condition. Therefore, these treatment differences may be affected by confounding by indication.

This study evaluated factors associated with mortality in neonatal HF and incorporated gestational age-based subgroup analyses to further characterize the influence of developmental maturity on clinical outcomes. Previous pediatric HF studies have generally included broad age ranges. A recent multicenter pediatric HF study reported that, within the neonatal subgroup, gestational age <34 weeks, modified Ross class III–IV at admission, complex congenital heart disease, moderate-to-severe valvular regurgitation, and infection were associated with mortality (5). Our study extends this evidence by focusing exclusively on neonates and incorporating detailed perinatal history, bedside organ-perfusion indicators, echocardiographic findings, respiratory-support requirements, and etiological stratification. From a practical standpoint, gestational maturity, perinatal history, urine output, and echocardiographic findings may provide complementary information for risk assessment. Neonates with multiple adverse features may warrant more intensive monitoring and earlier cardiovascular reassessment, although these variables should not be used as stand-alone prognostic criteria.

However, several limitations should be acknowledged. First, this was a single-center retrospective study with a relatively small sample size, which limited the precision and stability of the multivariable estimates. Second, treatment allocation was not randomized; therefore, confounding of treatment-related variables were susceptible to confounding by indication. Third, although the combined model showed good predictive efficacy, only 28 deaths occurred and four predictors were included, corresponding to approximately 7 events per predictor. Therefore, instability of the regression estimates and overfitting therefore cannot be excluded. Furthermore, the etiology-based and gestational-age subgroup analyses were exploratory and were limited by the small sample size and the few deaths, particularly in the <34-week and congenital-cause group. Because of the limited number of events, no additional covariates were included in the subgroup Firth regression models, and residual confounding remains possible. Therefore, this finding should be considered exploratory and larger studies are required to validate these results.

## Conclusion

Neonatal HF is associated with substantial mortality and is most commonly driven by congenital heart disease and other underlying conditions. Fetal distress and oliguria were associated with higher mortality, whereas higher LVEF was associated with lower mortality. Neonatal HF differs from HF in older children in etiology, pathophysiology, and clinical course, and outcomes also vary across gestational ages. These findings underscore the importance of early recognition, close monitoring, and targeted management of HF, particularly in preterm and high-risk neonates. Given the retrospective design, limited sample size, and heterogeneous etiologies, these findings should be interpreted cautiously and validated in larger multicenter studies.

## Data Availability

The original contributions presented in the study are included in the article/Supplementary Material, further inquiries can be directed to the corresponding authors.
