# Supplementary material for: Clinical characteristics and outcomes of neonatal heart failure: a single-center retrospective analysis
Source: Front Pediatr. 2026 Aug 26;14:1929001. doi: 10.3389/fped.2026.1929001 (PMC13553794; doi:10.3389/fped.2026.1929001)
Supplement: Supplementary file 1 [file Table1.docx]

Supplementary file 1. Comparison of clinical information in the congenital-cause group and non-congenital-cause group.

| Variables | non-congenital-cause group (n = 60) | congenital-cause group (n = 29) | Statistic | *P* |
| --- | --- | --- | --- | --- |
|  |  |  |  |  |
| gestational age (weeks), M (Q₁, Q₃) | 36.30 (33.60, 38.68) | 37.70 (35.70, 39.00) | Z=-1.68 | 0.094 |
| birth weight (grams), M (Q₁, Q₃) | 2735.00 (2022.50, 3125.00) | 3000.00 (2160.00, 3380.00) | Z=-1.46 | 0.144 |
| 1min Apgar scores, M (Q₁, Q₃) | 9.00 (5.75, 10.00) | 10.00 (7.00, 10.00) | Z=-1.38 | 0.168 |
| 5min Apgar scores, M (Q₁, Q₃) | 9.00 (7.00, 10.00) | 10.00 (8.00, 10.00) | Z=-1.03 | 0.303 |
| size of ASD, M (Q₁, Q₃) | 0.00 (0.00, 0.23) | 0.07 (0.00, 0.43) | Z=-2.10 | **0.036*** |
| size of VSD, M (Q₁, Q₃) | 0.00 (0.00, 0.00) | 0.07 (0.00, 0.50) | Z=-4.88 | **<.001*** |
| size of PDA, M (Q₁, Q₃) | 0.21 (0.00, 0.35) | 0.17 (0.00, 0.30) | Z=-1.19 | 0.235 |
| LVEF, M (Q₁, Q₃) | 57.00 (45.00, 65.00) | 62.00 (55.00, 65.00) | Z=-1.73 | 0.083 |
| BNP, M (Q₁, Q₃) | 26.00 (17.75, 36.00) | 34.00 (19.00, 42.00) | Z=-0.94 | 0.348 |
| pulmonary arterial pressure, M (Q₁, Q₃) | 46.50 (34.00, 55.00) | 53.00 (45.00, 62.00) | Z=-1.99 | **0.046*** |
| HF onset age (day), M (Q₁, Q₃) | 1.00 (1.00, 5.25) | 2.00 (1.00, 6.00) | Z=-0.97 | 0.333 |
| length of hospital stay, M (Q₁, Q₃) | 17.00 (10.00, 29.50) | 12.00 (4.00, 22.00) | Z=-2.19 | **0.028*** |
| boys, n(%) | 38 (63.33) | 15 (51.72) | χ²=1.09 | 0.296 |
| gestational diabetes, n(%) | 13 (21.67) | 7 (24.14) | χ²=0.07 | 0.793 |
| gestational hypertension, n(%) | 7 (11.67) | 5 (17.24) | χ²=0.15 | 0.696 |
| placental abnormalities, n(%) | 6 (10.00) | 1 (3.45) | χ²=0.43 | 0.512 |
| maternal immune-related diseases, n(%) | 6 (10.00) | 0 (0.00) | χ²=1.72 | 0.189 |
| fetal distress, n(%) | 20 (33.33) | 8 (27.59) | χ²=0.30 | 0.584 |
| intrauterine infection, n(%) | 15 (25.00) | 6 (20.69) | χ²=0.20 | 0.654 |
| EOS, n(%) | 22 (36.67) | 4 (13.79) | χ²=4.95 | **0.026*** |
| LOS, n(%) | 6 (10.00) | 1 (3.45) | χ²=0.43 | 0.512 |
| shock, n(%) | 31 (51.67) | 7 (24.14) | χ²=6.06 | **0.014*** |
| cardiomegaly, n(%) | 44 (73.33) | 19 (65.52) | χ²=0.58 | 0.447 |
| left ventricle enlargement, n(%) | 7 (11.67) | 5 (17.24) | χ²=0.15 | 0.696 |
| right ventricle enlargement, n(%) | 20 (33.33) | 12 (41.38) | χ²=0.55 | 0.458 |
| fully ventricle enlargement, n(%) | 17 (28.33) | 4 (13.79) | χ²=2.29 | 0.130 |
| ASD, n(%) | 19 (31.67) | 15 (51.72) | χ²=3.33 | 0.068 |
| VSD, n(%) | 4 (6.67) | 15 (51.72) | χ²=23.64 | **<.001*** |
| ventricular tachycardia, n(%) | 4 (6.67) | 0 (0.00) | χ²=0.77 | 0.381 |
| supraventricular tachycardia, n(%) | 9 (15.00) | 1 (3.45) | χ²=1.59 | 0.208 |
| PPHN, n(%) | 49 (81.67) | 27 (93.10) | χ²=1.24 | 0.266 |
| respiratory failure, n(%) | 51 (85.00) | 18 (62.07) | χ²=5.90 | **0.015*** |
| BPD, n(%) | 7 (11.67) | 1 (3.45) | χ²=0.77 | 0.382 |
| anemia, n(%) | 30 (50.00) | 4 (13.79) | χ²=10.86 | **<.001*** |
| oliguria, n(%) | 10 (16.67) | 3 (10.34) | χ²=0.22 | 0.637 |
| pulmonary hemorrhage, n(%) | 14 (23.33) | 3 (10.34) | χ²=2.13 | 0.144 |
| Intracranial hemorrhage, n(%) | 10 (16.67) | 1 (3.45) | χ²=2.05 | 0.152 |
| death, n(%) | 40 (66.67) | 21 (72.41) | χ²=0.30 | 0.584 |

*: Statistical significance (P<0.05) in test.

Abbreviations: ASD: atrial septal defect; VSD: ventricular septal defect; PDA: patent ductus arteriosus; LEVF: left ventricular ejection fraction; BNP: brain natriuretic peptide; HF: heart failure; EOS: Early-onset sepsis; LOS: Late-onset sepsis; PPHN: persistent pulmonary hypertension of the newborn; BPD: bronchopulmonary dysplasia; PPHN: persistent pulmonary hypertension of the newborn.
