# Supplementary material for: Clinical characteristics and outcomes of neonatal heart failure: a single-center retrospective analysis
Source: Front Pediatr. 2026 Aug 26;14:1929001. doi: 10.3389/fped.2026.1929001 (PMC13553794; doi:10.3389/fped.2026.1929001)
Supplement: Supplementary file 2 [file Table2.docx]

Supplementary file 2 Etiology-stratified associations of fetal distress, oliguria, and LVEF with mortality in neonates with heart failure

| Variables | subgroup | OR (95% CI) | *P* | *P* for interaction |
| --- | --- | --- | --- | --- |
| fetal distress | non-congenital-cause group | 11.84(3.859-44.604) | **<.001*** | 0.297 |
|  | congenital-cause group | 3.89 (0.739-22.138) | 0.108 |  |
| oliguria | non-congenital-cause group | 5.95 (1.529-27.665) | 0.010 | 0.926 |
|  | congenital-cause group | 5.26 (0.592-65.803) | 0.133 |  |
| LVEF, 5% increase | non-congenital-cause group | 1.01 (0.809-1.264) | 0.954 | **0.014*** |
|  | congenital-cause group | 0.57 (0.297-0.850) | 0.004 |  |

*: Statistical significance (P<0.05) in analysis.

Abbreviations: OR: Odds Ratio; CI: Confidence Interval; LEVF: left ventricular ejection fraction.
