# Supplementary material for: Clinical characteristics and outcomes of neonatal heart failure: a single-center retrospective analysis
Source: Front Pediatr. 2026 Aug 26;14:1929001. doi: 10.3389/fped.2026.1929001 (PMC13553794; doi:10.3389/fped.2026.1929001)
Supplement: Supplementary file 3 [file Table3.docx]

supplementary file 3. Gestational age–stratified associations of fetal distress, oliguria, and LVEF with mortality in neonates with heart failure

| Variables | subgroup | OR (95% CI) | *P* | *P* for interaction |
| --- | --- | --- | --- | --- |
| fetal distress | ≥34 weeks | 5.35 (1.63-18.41) | **0.006*** | 0.544 |
|  | <34 weeks | 10.92 (1.67-101.95) | **0.011*** |  |
| oliguria | ≥34 weeks | 5.19 (1.12-26.12) | **0.035*** | 0.792 |
|  | <34 weeks | 3.67 (0.54-42.51) | 0.192 |  |
| LVEF, 5% increase | ≥34 weeks | 0.82 (0.65-1.02) | 0.077 | 0.782 |
|  | <34 weeks | 0.88 (0.53-1.32) | 0.540 |  |

*: Statistical significance (P<0.05) in analysis.

Abbreviations: OR: Odds Ratio; CI: Confidence Interval; LEVF: left ventricular ejection fraction.
